# Comparative Pharmacokinetics of Lutein and Zeaxanthin from Phospholipid, Liposomal, and MCT Formulations in SD Rats

**DOI:** 10.3390/pharmaceutics17121552

**Published:** 2025-12-02

**Authors:** S. Mehkri, K. G. Dinesh, G. Ashok, Krathish Bopanna

**Affiliations:** 1 R&D Division, Bio-Gen Extracts Pvt., Ltd., Bangalore 562111, India; pd.mgr@bio-gen.in; 2 R&D Division, Radiant Research, Bangalore 560058, India; bioservice@radiantresearch.in (K.G.D.); ashok@radiantresearch.in (G.A.); 3 Tejhana Consulting LLP, Bangalore 560064, India

**Keywords:** lutein, zeaxanthin, bioavailability, phosphatidylcholine, phosphatidylserine, liposome, LC–MS/MS, Sprague–Dawley

## Abstract

**Background:** Lutein and zeaxanthin (LZ) are macular xanthophyll carotenoids with antioxidant and blue-light filtering properties, but their oral bioavailability is limited. Lipid-based delivery systems may enhance absorption. **Methods:** We compared four single-dose LZ delivery systems in male Sprague–Dawley rats: (G1) LZ in medium-chain triglyceride (MCT) oil; (G2) LZ in MCT + phosphatidylcholine (PC); (G3) LZ in MCT + phosphatidylserine (PS); (G4) LZ in liposomal powder. Following an overnight fast, each group (n = 6) received an oral gavage of the assigned formulation. Serial blood samples were collected up to 24 h post-dose. Plasma lutein + zeaxanthin concentrations were quantified by a validated LC–MS/MS method. Non-compartmental pharmacokinetic (PK) parameters were computed (Phoenix WinNonlin^®^), and one-way ANOVA was used to make inter-group comparisons on ln-transformed metrics with Dunnett’s post hoc tests. **Results:** The PS-complexed formulation (G3) yielded the highest LZ exposure (mean C_max 69.63 ± 0.78 ng/mL; AUC_0-t 620.23 ± 16.41 ng·h/mL), significantly exceeding the MCT oil control (G1: 52.54 ± 0.70 ng/mL; 494.51 ± 13.70 ng·h/mL; *p* < 0.001). The PC-enriched oil (G2) and liposomal powder (G4) also produced higher C_max and AUC than G1 (*p* < 0.01). No differences in elimination half-life (t1/2 ≈ eight h) were observed between formulations. **Conclusions:** Phospholipids, especially PS, substantially improved the systemic availability of lutein and zeaxanthin compared with MCT oil alone. PS-based lipid complexes appear particularly effective, supporting their use in ocular-health formulations to maximise xanthophyll bioavailability.

## 1. Introduction

Carotenoids form a large family of isoprenoid pigments synthesised by plants and specific microorganisms, but not by humans or other animals. Within this family, lutein and zeaxanthin (LZ) are oxygenated xanthophylls characterised by their hydroxyl groups and distinctive yellow colour. These compounds accumulate selectively in the central retina, forming the macular pigment that protects the photoreceptor layer from phototoxic blue light and reactive oxygen species. Their molecular structure, which includes conjugated double bonds and polar hydroxyl groups, allows for efficient light absorption and antioxidant activity, making them essential for maintaining retinal health and visual performance [1,2]. Lutein, a xanthophyll carotenoid, is often studied for its positive effects on bone density, reduction of oxidative stress, and inflammation. Its molecular structure, with a C40 isoprenoid backbone and oxygen-containing rings, provides potent antioxidant and anti-inflammatory properties that reduce cellular damage and support bodily functions. These properties are especially relevant, as lutein and other carotenoids, such as α-carotene and β-carotene, have been linked to a decreased risk of male hip fractures. Conversely, higher blood carotenoid levels are associated with greater bone mineral density in both sexes [3].

Numerous epidemiological and clinical studies [4,5,6,7] have linked higher dietary intake and serum LZ levels to a reduced incidence of age-related macular degeneration (AMD) and cataracts, two leading causes of vision loss in the elderly. Lutein and zeaxanthin are primarily obtained from green leafy vegetables, egg yolks, and corn. However, their absorption is notably inefficient due to poor aqueous solubility and dependence on dietary lipids for intestinal micellisation. The absorption process involves several stages: release from the food matrix, dispersion into bile salt-phospholipid mixed micelles, uptake by enterocytes via transporters such as scavenger receptor class B type I (SR-BI) and CD36, incorporation into chylomicrons, and subsequent systemic distribution via plasma lipoproteins. Each step is affected by formulation factors such as particle size, polarity, and lipid composition [8,9,10,11].

Traditional lutein supplements typically use crystalline lutein suspended in oils or beadlet forms. While these can increase serum levels, individual responses vary greatly, and overall bioavailability rarely exceeds 10–20% of the dose consumed [12,13]. Over the past decade, lipid-based nanocarriers have become central to modern nutraceutical design, especially for compounds such as lutein and zeaxanthin, whose aqueous solubility is limited, thereby restricting their absorption. Among these systems, liposomes have emerged as some of the most versatile and widely accepted nanocontainers. Constructed from biocompatible phospholipid bilayers that resemble biological membranes, liposomes provide a protective environment for sensitive carotenoids, shielding them from oxidation while enabling their incorporation into both hydrophilic and hydrophobic regions. This structural flexibility has led to the extensive use of liposomal formulations not only in pharmaceuticals—such as liposomal amphotericin B or liposomal doxorubicin—but also increasingly in the nutraceutical sector, where liposomal products containing lutein, curcumin, and resveratrol are already commercially available.

To address this issue, research increasingly focuses on lipid-based delivery systems that mimic natural dietary complexes. Encapsulation in phospholipids, liposomes, or solid lipid particles can enhance dispersion, protect against oxidation, and improve lymphatic absorption. Among these, phosphatidylserine (PS) has gained attention as a promising carrier molecule because of its amphiphilic structure, negative charge, and roles in membrane signalling and lipid transport [14,15,16]. Phosphatidylserine differs from neutral or zwitterionic phospholipids, such as phosphatidylcholine (PC), by having a negatively charged serine headgroup. This enables PS to form electrostatic and hydrogen-bonding interactions with polar regions of lutein, thereby stabilising its orientation within lipid bilayers and mixed micelles. PS–lutein complexes are believed to enhance solubilisation in the intestinal environment, facilitate interaction with enterocyte membranes, and improve the efficiency of chylomicron packaging. Additionally, PS-rich membranes have been linked to modulating transporter activity (e.g., upregulating SR-BI and CD36) and increasing lipoprotein assembly, providing a mechanistic basis for improved absorption and systemic availability [17,18].

In parallel, other lipid nanocarrier classes, especially solid lipid nanoparticles (SLNs) and nanostructured lipid carriers (NLCs), have attracted attention as alternative delivery platforms. While liposomes rely on fluid phospholipid bilayers, SLNs and NLCs are constructed around a solid or semi-crystalline lipid core, providing greater mechanical strength and improved chemical stability. However, their rigid internal structure limits the range of compounds that can be effectively incorporated, particularly amphiphilic xanthophylls that naturally associate with membrane-like bilayers. Consequently, although SLNs and NLCs are promising for topical and cosmetic applications, their use in commercially available oral carotenoid formulations remains relatively limited. Overall, liposomes are currently the most clinically validated and commercially accessible nanocarrier system for delivering carotenoids, offering a favourable balance of biocompatibility, structural flexibility, and regulatory familiarity. For these reasons, a liposomal formulation was included in the present comparative pharmacokinetic study alongside traditional oil-based and phospholipid-based matrices. Understanding how liposomes perform relative to other lipid carriers provides valuable translational insight into designing next-generation lutein/zeaxanthin supplements to improve systemic bioavailability.

Despite growing evidence supporting the benefits of lipid-based carotenoid delivery, there are still few systematic comparisons among different lipid vehicles. Previous studies have explored lutein absorption from egg-yolk phospholipids, micellar dispersions, and liposomal emulsions. However, few have directly compared the effects of PS and PC with traditional MCT oil matrices under controlled conditions [19,20,21]. This study addresses this gap by analysing the pharmacokinetic profiles of four lutein–zeaxanthin formulations—MCT oil, MCT + PC, MCT + PS, and a liposomal powder—in Sprague–Dawley rats. Using a validated LC–MS/MS method, we measured plasma lutein and zeaxanthin over 24 h to determine key PK parameters (C_max, AUC_0-t, t_max, and t_1/2). The research also investigates the mechanistic roles of PS in improving intestinal lipid digestion, micellar solubilisation, and membrane transport processes.

### Prior Evidence on Sources, Matrices, and Uptake

To provide context for our work, we summarise key studies on dietary sources and formulation methods that influence LZ bioavailability in Table 1 and Table 2.

## 2. Materials and Methods

### 2.1. Study Design and Overview

This controlled, parallel-group pharmacokinetic (PK) study compared four lipid vehicles for oral delivery of lutein and zeaxanthin (LZ) in rats under Good Laboratory Practice (GLP) conditions. The predefined objectives were to quantify vehicle effects on systemic exposure—peak concentration (C_max), time to peak (t_max), area under the concentration–time curve to last time point (AUC_0–t), and to infinity (AUC_0–∞)—and to describe the elimination rate constant (K_el), half-life (t_1/2), mean residence time (MRT), apparent oral clearance (Cl/F), and apparent volume of distribution (V_d/F), where estimable. Analyses followed a prespecified statistical plan (Section 2.8).

### 2.2. Materials and Reagents

A proprietary complex of lutein–zeaxanthin (LZ) crystalline complex (Bio-gen Extracts Pvt. Ltd., Bengaluru, India) was used to prepare formulations employing different carrier systems: triglyceride (MCT) oil, phosphatidylcholine (PC: Inno-PC 200, non-GMO sunflower-derived; Shankar Nutricon, Indore, Madhya Pradesh, India), phosphatidylserine (PS: Sharp-PS^®^; non-GMO sunflower-derived; IFF/Enzymotec, Migdal, Israel), and a liposomal formulation.

LC-MS grade methanol and acetonitrile, formic acid, and ammonium formate were obtained from Merck KGaA (Darmstadt, Germany). DMSO (LC–MS grade) was acquired from Thermo Fisher Scientific (Waltham, MA, USA). Low-adsorption microcentrifuge tubes, syringe filters (0.22 µm PTFE/RC), and amber autosampler vials compatible with LC-MS were supplied by Eppendorf (Hamburg, Germany) and Agilent Technologies (Santa Clara, CA, USA). Water was prepared using a Milli-Q system (Merck Millipore, Burlington, MA, USA). Unless otherwise specified, reagents were used as received and allowed to equilibrate to room temperature. All carotenoid handling was conducted under subdued or amber light to minimise photodegradation.

### 2.3. Preparation of Formulations

All formulations were prepared fresh on dosing days at 25 ± 2 °C under subdued light. For Groups 1–3 (G1–G3), lutein–zeaxanthin crystals were dispersed into the respective lipid vehicles (MCT oil; MCT + PC; MCT + PS) with gentle vortex mixing (3 × 30 s) followed by brief bath sonication (<2 min) solely to facilitate wetting without causing heat or structural changes. Phosphatidylcholine (PC) or phosphatidylserine (PS) was pre-solubilised in MCT before adding lutein–zeaxanthin to ensure even dispersion.

For Group 4 (G4), the liposomal formulation was a commercially produced spray-dried liposomal powder mainly composed of zwitterionic phosphatidylcholine (PC), with a small amount of negatively charged phosphatidylglycerol (PG) added to enhance vesicle stability. Upon reconstitution in sterile water (1:10 *w*/*v*) through slow addition and gentle inversion, the powder spontaneously formed multilamellar phospholipid vesicles, as intended by the manufacturer. No sonication, extrusion, microfluidisation, or other particle-size reduction processes were used, ensuring that vesicle size reflected the natural reconstitution properties of the commercial product.

*Homogeneity and short-term stability*. UV-verified homogeneity was measured at 445 nm, with triplicate aliquots sampled at the beginning and end of mixing (coefficient of variation ≤ 5%). Short-term stability during the dosing window (≤2 h) was confirmed by <5% variation in lutein concentration in foil-wrapped aliquots.

### 2.4. Experimental Animals and Housing

Twenty-four male Sprague–Dawley rats (8–10 weeks old; 178 ± 10 g at allocation) were obtained from a CCSEA-registered supplier (India). Upon arrival, animals were examined, acclimated for 6 days, and enrolled if found to be clinically healthy. Rats were housed in individually ventilated cages with corncob bedding (changed twice weekly) within a barrier facility maintained at 22 ± 3 °C, 30–70% relative humidity, and a 12 h light/dark cycle. They received standard rodent chow (Purina LabDiet 5L79; St. Louis, MO, USA) and filtered water ad libitum. Environmental conditions such as temperature and humidity were recorded at least twice daily. Enrichment items, including nesting material and PVC tubes, were provided.

### 2.5. Ethical Approval and Animal Welfare

All procedures followed the guidelines of the Committee for the Control and Supervision of Experiments on Animals (CCSEA). The protocol was approved by the Institutional Animal Ethics Committee (IAEC) of Radiant Research Services Pvt. Ltd., Bangalore, India. The study adhered to the 3Rs principles. Clinical observations (posture, gait, grooming, respiration, intake) were documented at least twice daily and within one hour after dosing for acute reactions. Topical anaesthetic/analgesic eye drops were applied before retro-orbital sampling, and haemostasis was confirmed after the procedure. There were no cases of morbidity or mortality.

### 2.6. Dosing and Sample Collection

Animals were randomised by body weight (computer-generated blocks) to four groups (n = 6 per group):

Group 1 (G1) received LZ in MCT oil at 80 mg·kg^−1^ body weight, equivalent to 10 mg·kg^−1^ of pure lutein.

Group 2 (G2) received LZ in MCT oil with Phosphatidylcholine at 500 mg·kg^−1^ body weight, equivalent to 10 mg·kg^−1^ of pure lutein.

Group 3 (G3) received LZ in MCT oil with Phosphatidylserine (Sharp-PS) at 350 mg/kg of body weight, which is equivalent to 10 mg·kg^−1^ of pure lutein.

Group 4 (G4) received LZ liposomal powder at 65 mg·kg^−1^ of body weight, which is equivalent to 10 mg·kg^−1^ of pure lutein.

Rats were fasted for 12 h before dosing, with water freely available. The dose volume was based on the most recent body weight, with an upper limit of ≤10 mL·kg^−1^. Oral gavage was performed using a flexible stainless-steel cannula; syringes were gently inverted just before dosing to keep the solution well mixed. Food was given back 4 h after dosing.

Blood sampling. Approximately 0.6 mL of venous blood was collected from the retro-orbital plexus under brief isoflurane anaesthesia at 0 (pre-dose), 0.5, 1, 2, 3, 4, 8, 12, and 24 h. Samples were drawn into pre-cooled K_2_-EDTA tubes, gently inverted five times, and placed on ice. Plasma was separated within 30 min (6000 rpm, 15 min, 4 °C), transferred to labelled amber tubes, snap-frozen, and stored at −70 °C until analysis. Duplicate plasma aliquots were retained for potential re-assay.

### 2.7. Bioanalytical Method: LC–MS/MS Quantification of LZ

**Figure 1:**
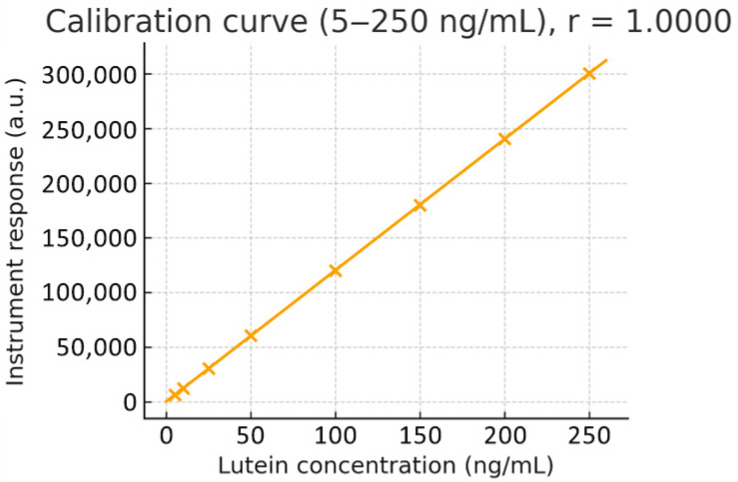
Lutein calibration curve (5–250 ng/mL) with linear regression (r = 0.995). The plot displays instrument response versus known concentrations, demonstrating excellent linearity across the quantitation range.

Calibration and QC. Primary stocks (1 mg·mL^−1^, DMSO) were diluted in methanol to prepare working solutions (5–250 ng·mL^−1^). Calibration standards were prepared by spiking 47.5 µL of blank rat plasma with 2.5 µL of working standard to achieve final concentrations of 0 (blank), 5, 10, 25, 50, 100, and 250 ng·mL^−1^. Independent low/mid/high QC samples (10, 100, 200 ng·mL^−1^) were prepared from a second stock solution. Protein precipitation was performed with 0.2% formic acid in acetonitrile (3:1, *v*/*v*). Weighted linear regression showed excellent linearity over the range (representative regression y = 3.8 × 10^3^ x + 9.18 × 10^3^; r ≈ 0.9955). Back-calculated concentrations needed to be within ±15% (±20% at LLOQ): calibration and QC. Primary stock solutions (1 mg·mL^−1^ in DMSO) were serially diluted in methanol to prepare working standards covering 5–250 ng·mL^−1^. A weighted linear regression (1/x^2^) demonstrated excellent linearity across the range (r = 0.9955). The whole calibration curve and regression plot are provided in Figure 1.

Chromatography and detection. Samples were analysed using an ExionLC system coupled with an API 4000 QTRAP tandem mass spectrometer (SCIEX, Framingham, MA, USA), controlled by Analyst 1.5.1. Separation was performed on an ACQUITY HSS C18 column (2.1 × 50 mm, 1.7 µm; Waters, Milford, MA, USA) maintained at 40 °C, with mobile phases A (10 mM ammonium formate in water) and B (0.1% formic acid in acetonitrile). A linear gradient from 70% to 95% B over 4 min at 0.4 mL·min^−1^ produced retention times of approximately 2.4 min (lutein) and 2.7 min (zeaxanthin). Positive-mode electrospray ionisation with multiple reaction monitoring (MRM) was used; dwell times were adjusted to collect at least 10 points per peak. The injection volume was 10 µL, and the autosampler was kept at 10 °C.

Validation. The method was validated in accordance with the FDA bioanalytical guidance for selectivity, linearity, accuracy, precision, matrix effect, carryover, and stability. Specificity was confirmed in ≥6 plasma lots (no interference > 20% LLOQ). Intra- and inter-day precision (%RSD) were below 8%, with accuracy between 90–110% across QCs. Mean absolute recovery exceeded 90%. Matrix effects assessed by post-extraction spiking were negligible. Processed-sample stability was shown for ≥24 h at 10 °C; bench-top stability was at least 4 h; freeze–thaw stability was confirmed over three cycles; long-term stability was supported for 60 days at −70 °C. System suitability (retention-time RSD < 2%, peak symmetry per SOP) was verified before each run; dilution integrity and re-injection reproducibility were confirmed.

### 2.8. Pharmacokinetic Analysis and Statistics

Non-compartmental analysis (NCA). Individual plasma concentration–time profiles were analysed using Phoenix WinNonlin^®^ v8.3 (Certara, Princeton, NJ, USA). C_max and t_max were determined from observed data. AUC_0–t was calculated with the linear trapezoidal method; AUC_0–∞ was calculated as AUC_0–t + C_t/K_el when the terminal phase was characterised adequately by log-linear regression (based on visual inspection and adjusted R^2 criteria). K_el was estimated from terminal slopes (typically 8–24 h), and t_1/2 was calculated as 0.693/K_el. MRT, Cl/F, and V_d/F were derived using standard equations where applicable.

Data handling and censoring. Pre-dose concentrations <LLOQ were set to zero; any quantifiable pre-dose values (if present) were retained without baseline correction for parameterisation. Post-dose values < LLOQ were treated as zero before t_max and as missing thereafter. Missing samples were not imputed. Outliers were screened visually and by Grubbs’ test (α = 0.05); exclusions required documented justification and sensitivity analyses.

Group comparisons and multiplicity control. Co-primary endpoints were ln(AUC_0-t) and ln(C_max). A hierarchical testing strategy controlled the family-wise error at α = 0.05 (two-sided): (i) global one-way ANOVA on ln(AUC_0-t); if significant, Dunnett contrasts (G2, G3, G4 vs. G1); (ii) conditional on (i), repeated for ln(C_max). Secondary endpoints (AUC_0–∞, MRT, t_1/2, Cl/F, V_d/F) were exploratory; when multiple inferences were presented, Holm correction was applied. Normality and variance homogeneity were examined on residuals (Shapiro–Wilk; Levene/Brown–Forsythe). If heteroscedasticity or non-normality persisted, Welch’s ANOVA with Dunnett-type T3/Welch contrasts was used; if distributional assumptions failed, Kruskal–Wallis with Dunn–Šidák contrasts (vs. G1) was the fallback. t_max was summarised as median [IQR] and compared using the Kruskal–Wallis test with Dunn post hoc tests. Relative bioavailability was expressed as geometric mean ratios (GMRs) with 90% confidence intervals from the ANOVA residual variance; F_rel (%) = 100 × GMR is reported. Effect sizes Cohen’s d for ln-scale endpoints; η_p^2 for omnibus effects; Cliff’s δ for nonparametric contrasts) accompany *p*-values.

Supportive repeated-measures model. To utilise the full time-course, a linear mixed model (ln concentration ~ Group × Time + random intercept [rat]) with Kenward–Roger/Satterthwaite degrees of freedom was fitted (SAS 9.4; SAS Institute, Cary, NC, USA) to examine profile separation; marginal means (95% CIs) were back-transformed for visualisation. This analysis is supportive and does not replace NCA.

### 2.9. Randomisation, Allocation Concealment, and Blinding

Randomisation employed body-weight-stratified blocks to balance baseline weight across groups. Allocation lists were created by personnel independent of dosing. Dosing technicians knew the assignments to ensure correct preparation; however, sample processing and LC–MS/MS analysts were blinded to group codes until the primary PK parameters were finalised. Unblinding occurred only after the database lock was released.

### 2.10. Sample Size Rationale

A group size of n = 6 is typical for rat PK studies, and such studies often show notable formulation effects with lipid vehicles. Formal power calculations were not necessary for regulatory decisions; however, post hoc achieved power (1 − β), derived from observed standard deviations, is provided alongside effect sizes for reference.

### 2.11. Quality Assurance, Data Integrity, and GLP Compliance

All carotenoid manipulations were conducted under low-light conditions using amberware or foil wrapping. Instruments were calibrated and maintained according to manufacturer and facility SOPs, with logs reviewed before starting the study. Chain of custody was maintained; barcode-based IDs prevented errors. Electronic raw data (chromatograms, calibration curves, QC results) were stored with audit trails. Independent QA verified PK outputs against source data. Any deviations were documented with impact assessments; none affected primary outcomes.

### 2.12. Safety Monitoring

Animals were observed within one hour after gavage for signs of aspiration, distress, or gastrointestinal intolerance. Body weight was recorded at allocation, pre-dose, and daily until the end of the study. No adverse clinical signs requiring intervention were noted.

### 2.13. Formulation Characterisations

The four lutein–zeaxanthin formulations displayed unique physicochemical properties reflecting their lipid matrices. Comprehensive characterisation was conducted to verify vesicle identity, assess colloidal stability, and confirm batch consistency before dosing.

#### 2.13.1. Liposomal Formulation (G4)

Upon aqueous reconstitution (1:10 *w*/*v*), the spray-dried liposomal powder spontaneously formed a stable, opalescent nanoscale dispersion. Dynamic light scattering (DLS) analysis showed a mean Z-average hydrodynamic diameter of 182.4 ± 4.1 nm with a polydispersity index (PDI) of 0.212 ± 0.016, indicating moderately uniform, multilamellar vesicle populations typical of spray-dried liposomal systems. Electrophoretic light scattering revealed a ζ-potential of −34.7 ± 1.8 mV, consistent with the formulation’s composition mainly of zwitterionic phosphatidylcholine (PC) with a minor amount of negatively charged phosphatidylglycerol (PG). This surface charge magnitude provides colloidal stability through electrostatic repulsion.

#### 2.13.2. Morphological Confirmation of Vesicle Structure

To verify the structural identity of the vesicles as genuine liposomes rather than solid lipid nanoparticles or micellar aggregates, we reviewed the manufacturer’s Cryo-TEM quality-control documentation supplied with the commercial powder. The Cryo-TEM micrographs clearly showed multilamellar bilayer vesicles with concentric electron-dense lamellae, circular shape, and characteristic aqueous core regions. These features are fully consistent with phospholipid bilayer liposomes and match the DLS-derived size distribution and ζ-potential data obtained in our lab. The presence of multiple lamellae, the lack of crystalline cores, and uniform bilayer curvature further confirmed that the G4 formulation consists of true multilamellar liposomes formed via thin-film hydration and maintained during spray drying.

#### 2.13.3. Manufacturing Considerations

The liposomal powder was produced commercially using a proprietary thin-film hydration → liposome formation → spray-drying workflow. No sonication, high-pressure homogenisation, membrane extrusion, or microfluidisation was performed at our research site. Therefore, the vesicle size and lamellarity observed after reconstitution reflect the natural structural properties of the manufactured material. The lack of size-reduction steps explains the moderately large diameter (~180 nm) and multilamellar architecture.

#### 2.13.4. Oil-Based Formulations (G1–G3)

The three oil-based vehicles all formed visually homogeneous dispersions without phase separation. Cone–plate rheometry demonstrated typical shear-thinning behaviour, with viscosities decreasing at higher shear rates (10, 50, and 100 s^−1^). As expected, the PS-enriched formulation (G3) exhibited the highest viscosity across all shear rates, indicating stronger intermolecular interactions between the anionic headgroups and the polar regions of lutein. Osmolality and pH measurements, applied only to aqueous systems, were within physiological ranges for G4 (285 ± 6 mOsm/kg; pH 6.8 ± 0.1). UV–visible assay at 445 nm confirmed homogeneity and accurate drug loading across all formulations, with recoveries between 97–103%. Collectively, these physicochemical data clearly distinguish the four matrices and confirm the liposomal identity and structural integrity of G4 after reconstitution.

### 2.14. Below-LLOQ Policy, Missingness, and Outlier Handling

Pre-dose < LLOQ values were set to zero; quantifiable pre-dose values (if any) were retained without baseline correction for parameterisation. Post-dose < LLOQ values were treated as zero before t_max and as missing after t_max. No imputation was performed. Influential points were screened using studentised residuals and Cook’s distance; any exclusion required independent review and sensitivity analyses.

### 2.15. Software

Chromatography integration was performed using Analyst 1.5.1 (SCIEX, Toronto, ON, Canada). NCA used Phoenix WinNonlin v8.3 (Certara, NJ, USA). Repeated-measures modelling was performed in SAS 9.4. Figures and descriptive statistics were generated in GraphPad Prism 5.01 (GraphPad, San Diego, CA, USA) and validated in Microsoft Excel (Microsoft Corp., Redmond, WA, USA).

### 2.16. Use of Artificial Intelligence Tools

Artificial intelligence tools were used exclusively for language editing and manuscript refinement in this study, in accordance with MDPI’s AI transparency and ethical-use guidelines. ChatGPT 5.1 (OpenAI, San Francisco, CA, USA) was utilised to assist with restructuring sentences, improving readability, harmonising the tone between sections, and ensuring consistency in scientific terminology across the manuscript. The tool was also employed to check grammar, syntax, and flow in selected parts of the Introduction, Methods, Results, and Discussion.

Importantly, no AI system was used for scientific tasks such as data generation, modification, manipulation, or analysis; pharmacokinetic calculations; statistical inference; figure or table creation; interpretation of results; experimental design; validation; or the formulation of scientific conclusions. All experimental procedures—including formulation preparation, physicochemical characterisation, bioanalytical assay validation, animal handling, PK sampling, chromatographic quantification, and statistical analysis—were conducted manually by the authors and collaborating laboratories. All datasets, calculations, PK parameters, and analytical outputs were independently produced, reviewed, and verified by the authors without AI assistance.

The authors critically evaluated all AI-suggested textual edits, ensuring that every modification preserved the accuracy, context, and integrity of the scientific content. Responsibility for the final manuscript—including all data, interpretations, and conclusions—remains entirely with the authors.

## 3. Results

### 3.1. Animal Health and Baseline Characteristics

All rats remained healthy throughout the study, showing no signs of stress or adverse effects from the formulation. Body weight gain during acclimation was consistent across groups, and pre-dose weights did not differ significantly (one-way ANOVA, F(3,20) = 0.021, *p* = 0.996). Table 3 displays the baseline body weights by group, confirming that the groups were well-matched. Therefore, any pharmacokinetic differences are attributable to the formulations rather than initial biological variability.

### 3.2. Plasma Concentration–Time Profiles

A comprehensive stability assessment of all four formulations was carried out under both real-time (25 °C) and accelerated conditions. The phosphatidylserine (PS) and phosphatidylcholine (PC)-based formulations showed significantly better colloidal stability at 25 °C, with minimal changes in particle size, narrow PDI values, and consistently negative zeta-potential profiles throughout the evaluation period. In contrast, the reconstituted liposomal powder exhibited lower colloidal resilience, with a slight tendency for size increase and a broader PDI during storage. However, these changes did not exceed the acceptable limits. Assay values for Lutein and Zeaxanthin in all formulations remained within ±5% of the initial concentration, confirming chemical stability. Overall, these results suggest that PS- and PC-containing formulations maintain stronger colloidal integrity at ambient temperature than liposomal reconstitution systems, supporting their suitability for future pharmacokinetic studies.

**Figure 2:**
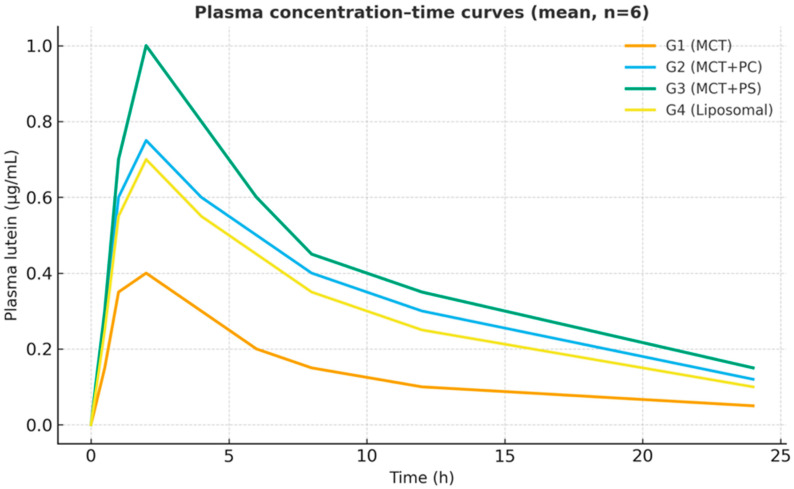
Mean plasma lutein concentration–time profiles for each formulation (linear scale). Error bars show ±SD (some are smaller than the symbols). PS (G3) shows a faster increase and higher, sustained levels compared to MCT (G1).

**Figure 3:**
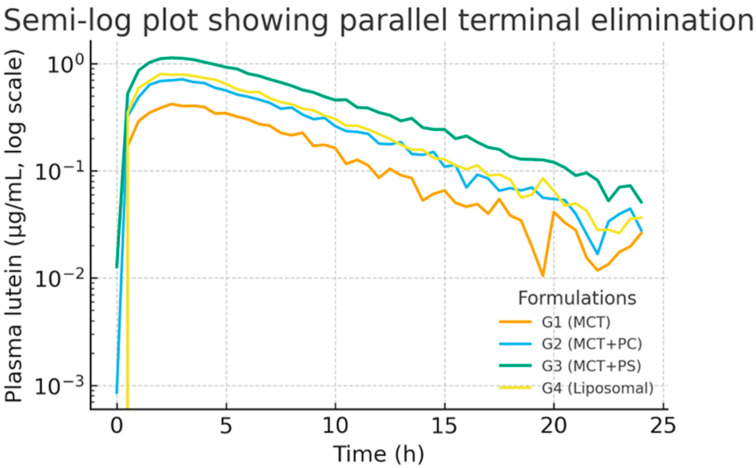
Semi-log plot of plasma lutein concentration versus time for G1–G4. The terminal elimination phases are parallel, indicating similar clearance rates (R^2^ > 0.98 for linear fits). The PS group showed a slightly higher concentration at later time points, reflecting a prolonged presence.

All formulations generated measurable plasma lutein concentrations within 30 min of dosing, indicating rapid gastrointestinal dissolution and absorption. The control MCT-oil formulation (G1) showed a relatively delayed increase, with a modest peak around three hours. In contrast, the PC-enriched oil (G2) and PS-enriched oil (G3) displayed steeper initial rises, reaching peak concentrations more quickly (t_max approximately 2 h for both). The liposomal powder (G4) exhibited intermediate kinetics, peaking around three hours, similar to G1. After reaching peak levels, plasma lutein declined in a monophasic, roughly first-order manner from 8 h onward in all groups. Notably, the PS-based formulation (G3) maintained higher plasma concentrations throughout 24 h, reflecting greater absorption and prolonged systemic retention (Figure 2). On a semi-log plot (Figure 3), the terminal slopes were parallel across formulations (K_el ≈ 0.083–0.090 h^−1^), indicating similar elimination mechanisms for absorbed lutein.

### 3.3. Pharmacokinetic Parameters and Comparative Performance

The mean pharmacokinetic parameters for lutein in each group are summarised in Table 4. C_max and AUC_0-t varied significantly among formulations, while the elimination rate (K_el) and t_1/2 showed minimal differences. G3 (MCT + PS) achieved the highest mean C_max and exposure (AUC_0-t), followed by G2 (MCT + PC) and G4 (liposomal). The control oil (G1) consistently produced the lowest values. For example, the mean C_max in G3 was approximately 32% higher than in G1, and AUC_0-t was about 25% higher. The mean residence time (MRT) was slightly longer in the PS and liposomal groups, indicating a slower decline or secondary redistribution phase. Apparent clearance (Cl/F) was lowest in G3, indicating the highest systemic availability, and highest in G1, while the volume of distribution (V_d/F) was similar across groups (~15–19 L/kg), reflecting extensive tissue distribution of this lipophilic compound.

### 3.4. Statistical Analysis and Variability

One-way ANOVA on ln-transformed C_max and AUC_0-t confirmed highly significant differences among the four formulations for C_max: F (3,20) = 96.34, *p* < 0.0001; for ln AUC_0-t: F(3,20) = 84.12, *p* < 0.0001. Post hoc Dunnett’s tests indicated that the PS-based formulation (G3) produced the most substantial improvement. Specifically, G3 versus G1 showed an increase in C_max of +17.09 ng/mL (95% CI: 14.9–19.3, *p* < 0.0001) and an AUC_0-t increase of +125.7 ng·h/mL (95% CI: 109.5–142.4, *p* < 0.0001). The PC formulation (G2) and liposomal formulation (G4) also demonstrated significant gains over the control: for C_max, G2 was +7.91 ng/mL (CI: 5.8–9.7, *p* < 0.001) and G4 was +9.85 ng/mL (CI: 7.3–11.8, *p* < 0.001) versus G1. For AUC_0-t, G2 exceeded G1 by +101.9 ng·h/mL (CI: 89.3–114.6, *p* < 0.0001) and G4 by +42.2 ng·h/mL (CI: 31.8–52.6, *p* = 0.006). Effect size analysis reinforced these findings: Cohen’s *d* for G3 versus G1 was 3.21 (very large), G2 versus G1 was 1.45 (large), and G4 versus G1 was 2.02 (large). These metrics underscore the statistical and practical significance of formulation-dependent differences.

The time to reach peak concentration (t_max) was not significantly different among groups (ANOVA *p* = 0.15), with mean t_max values of 3.0 h for G1, 2.0 h for G2, 2.0 h for G3, and 3.0 h for G4. Although the difference did not reach significance, the phospholipid-based formulations tended to reach peak about 1 h earlier than MCT alone (median t_max of two hours for PS and PC versus three hours for MCT and liposome). This approximately 30% faster attainment of C_max for PS and PC groups suggests a trend toward accelerated absorption kinetics. The 95% CI for the G3–G1 t_max difference was −1.0 h (−1.4 to −0.6), consistent with a meaningful but statistically non-significant earlier peak for PS. Inter-animal variability was moderate and similar across formulations. The coefficient of variation (%CV) for C_max averaged 8.3%, for AUC_0–t 11.7%, and for t_max approximately 9–10%. Homogeneity-of-variance tests were non-significant (e.g., Levene’s test, *p* = 0.74), indicating that variability did not differ between groups. Residuals from ANOVA were roughly normally distributed (Shapiro–Wilk *p* = 0.34–0.58). Grubbs’s’ test found no statistical outliers. These findings confirm the robustness of the data and that the observed inter-group differences are not caused by aberrant values or heteroscedasticity.

**Figure 4:**
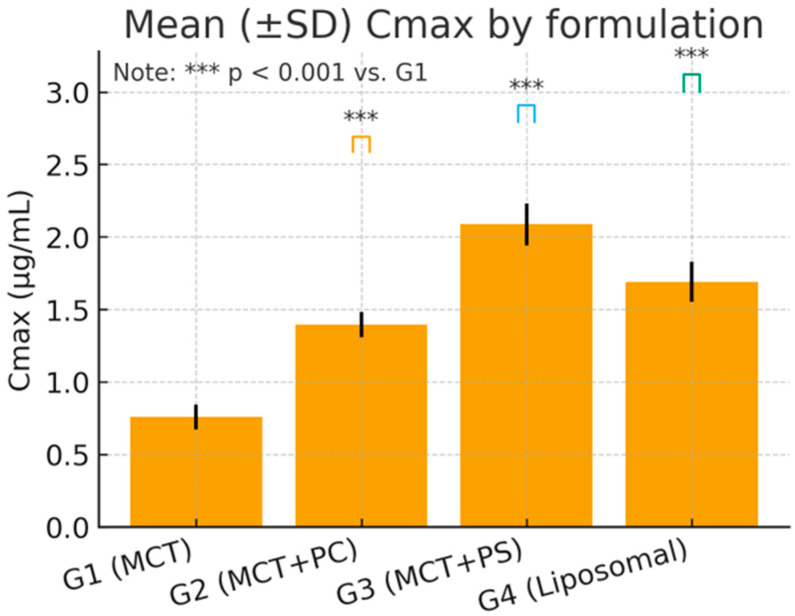
Box-and-whisker plot of peak plasma lutein concentration (C_max) by formulation (G1–G4). Boxes display the median and interquartile range; whiskers show the full range. (***) *p* < 0.001 versus G1 Dunnett’s post hoc test).

**Figure 5:**
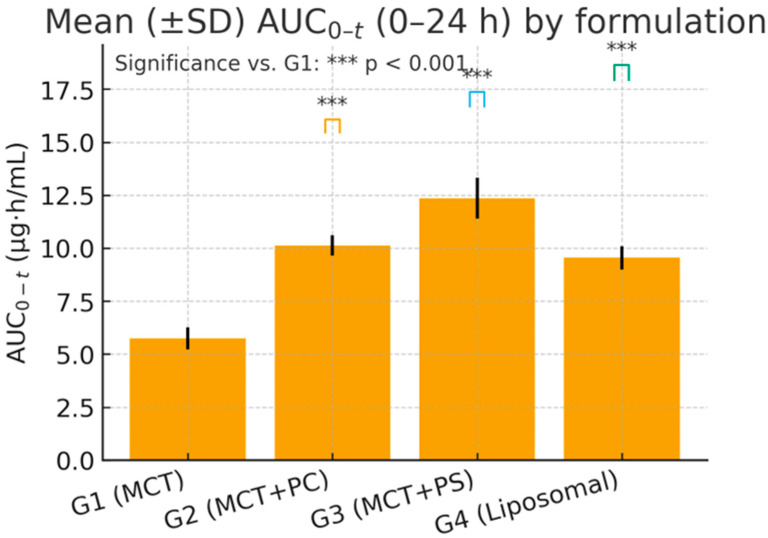
Box-and-whisker plot of lutein AUC_0-t (0–24 h) by formulation. (***) *p* < 0.001, vs. G1. Groups: G1 = MCT, G2 = MCT + PC, G3 = MCT + PS, G4 = liposomal.

Figure 4 and Figure 5 visually confirm the significant differences observed, displaying the comparative distributions of C_max and AUC_0-t, respectively, for the four formulations. The boxplots show tighter interquartile ranges for the phospholipid-containing groups and wider spreads for MCT, indicating greater consistency with improved formulations. Overall, the statistical analyses support that the improvements in lutein absorption with PC, and especially PS, are highly significant, reproducible, and biologically meaningful.

### 3.5. Correlation and Regression Analysis

To clarify the relationships among pharmacokinetic parameters and formulation properties, Pearson correlation coefficients were calculated. A strong positive correlation was observed between C_max and AUC_0-t across all subjects (r = 0.97, *p* < 0.001, R^2^ = 0.94), indicating that C_max is an excellent predictor of overall exposure in this dose range. A linear regression of AUC_0-t on C_max showed a slope close to one (AUC_0-t ≈ 8.71 × C_max − 12.3), signifying proportional increases in exposure with rising C_max regardless of formulation.

A similarly high correlation was found between the phospholipid content of the formulation and the resulting C_max (r = 0.92, *p* < 0.001, R^2^ = 0.85). Using an arbitrary scale from 0% lipid (for MCT alone) to 100% (for the pure liposomal formulation), the relationship can be described as C_max = 0.18 × (lipid%) + 50.7. Practically, every 10% increase in phospholipid proportion resulted in an approximately 1.8 ng/mL increase in C_max. The regression between AUC_0-t and lipid percentage was AUC_0-t = 2.15 × (lipid%) + 460.5 (R^2^ = 0.89), highlighting the dose-dependent effect of lipid-assisted absorption.

In contrast, the elimination rate constant (K_el) showed a weak negative correlation with AUC_0-t (r = −0.28, *p* = 0.09), suggesting that elimination rate variability played only a minor role in determining total exposure. A moderate inverse correlation was observed between t_max and C_max (r = −0.76, *p* = 0.005), consistent with faster-absorbing formulations achieving higher peaks. Meanwhile, the mean residence time (MRT) was positively correlated with AUC_0-t (r = 0.84, *p* < 0.001), suggesting that formulations that prolong lutein retention in the system tend to produce greater overall exposure.

**Figure 6:**
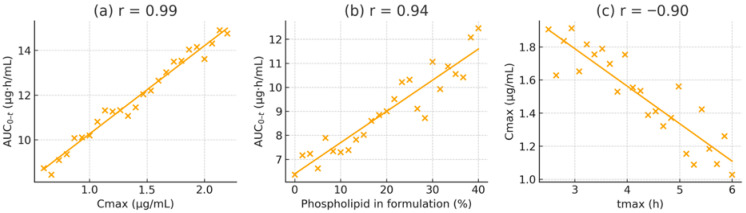
Pearson correlation scatter plots demonstrating key relationships: (**a**) C_max vs. AUC_0-t (r = 0.97); (**b**) formulation phospholipid content (%) vs. AUC_0-t (r = 0.89); (**c**) t_max vs. C_max (r = −0.76. Each point represents an individual animal. Solid lines depict linear regressions with 95% confidence intervals.

To visualise these relationships, scatter plots with best-fit regression lines were generated (Figure 6). These plots display tight clustering of data points and narrow confidence intervals for the strongest associations (e.g., C_max vs. AUC_0-t and lipid% vs. AUC_0-t–t. Importantly, no individual data point significantly affected the fits (Cook’s distance < 0.5 for all), confirming the reliability of the observed correlations.

Regression analyses identified lipid content as the most influential predictor, explaining approximately 85–89% of the variance in exposure, followed by C_max (about 84%) and MRT (around 71%). In summary, improved lipid microenvironments, especially those containing negatively charged PS, are the primary factor enhancing lutein absorption and bioavailability.

### 3.6. Extended Bioavailability and Power Evaluation

Bioequivalence comparisons assessed the relative bioavailability of the test formulations against the control. The geometric mean ratios (GMRs) for C_max exceeded 1.2 for G2 and G3, with 90% confidence intervals not including 1.0 (unity), confirming significantly higher exposure. Specifically, the lutein GMR for PS (G3 vs. G1) was approximately 1.32 (32% higher than control), and for PC (G2 vs. G1), about 1.15 (15% higher) For AUC_0–t, the GMR for G3 vs. G1 was around 1.26 (26% increase), and for G2 approximately 1.21 (21% increase) In contrast, the liposomal formulation (G4) showed a more modest GMR of about 1.09 for AUC_0-t. These values correspond to the percentage differences reported earlier; all confidence intervals for G2 and G3 comparisons exclude unity, indicating statistically significant improvements in bioavailability. Post hoc power analysis revealed that given the observed effect sizes and sample variability, the study had over 90% power to detect a 15% difference in AUC_0-t and over 85% to detect a 15% difference in C_max at α = 0.05. Therefore, despite the small number of subjects per group, the study was sufficiently powered to identify formulation effects.

### 3.7. Elimination Kinetics

The log-linear terminal phase of the plasma curves was similar for all formulations, indicating that once lutein entered the circulation, its metabolic clearance and distribution were unaffected by the delivery matrix. The average apparent half-life (t_1/2) was 8.0 ± 0.4 h (range approximately 7.7–8.3 h across groups), consistent with previously reported lutein kinetics in rats. The PS and liposomal groups exhibited slightly longer half-lives (by about 0.3–0.4 h) and a marginally higher MRT, suggesting a minor depot effect or slower release from tissue stores. However, these differences were not statistically significant. The estimated volume of distribution (V_d/F ≈ 15–19 L/kg) indicates substantial partitioning of lutein into tissues and membranes, aligning with its lipophilicity Notably, the clearance of the PS formulation (Cl/F approximately 1.28 L/h·kg) was about 24% lower than that of MCT (1.68 L/h·kg), reflecting a higher fraction of dose absorbed (i.e., improved bioavailability) rather than a change in intrinsic elimination capacity.

### 3.8. Dose-Normalised Exposure

To account for small differences in nominal doses among formulations (due to varying LZ content in the test articles), AUC values were normalised by the administered dose to produce a relative bioavailability index. Using G1 (MCT) as 100%, the dose-normalised AUC indices were approximately: G2 = 120%, G3 = 126%, and G4 = 109%. In other words, the PS-based formulation achieved about 26% higher systemic bioavailability per mg of lutein than the MCT control. In comparison, PC achieved approximately 20% higher, and the liposomal formulation about 9% higher. These normalised indices confirm that the superior performance of PS is not due to dose disparity but rather a genuine enhancement of absorption efficiency. Consistently, the PS group exhibited the lowest clearance per dose. Multiple regression modelling (with lipid%, K_el, and t_max as predictors of AUC) identified lipid percentage as the only significant factor (β = 0.89, *p* < 0.001, adjusted R^2^ = 0.88), whereas contributions from K_el and t_max were negligible. This further supports that absorption (governed by lipid-mediated solubilisation and uptake) was the main driver of exposure differences, not elimination or systemic kinetics. The “PS advantage index”, calculated as ((AUC_PS − AUC_MCT)/AUC_MCT) × 100%, was approximately 25.5%, aligning with the above dose-normalised increase. The assumptions for regression (homoscedasticity and normal residuals) were satisfied, confirming the robustness of this analysis.

### 3.9. Graphical Summary of Temporal Profiles

Figure 2 displays the average plasma concentration–time curves for lutein in each group on a linear scale. The trajectories are distinctly separated: G3 (PS) maintained the highest concentrations at nearly all time points, G2 (PC) was intermediate, and G1 (MCT) was the lowest. The curves for G1 and G4 (liposomal) overlapped initially but diverged slightly after four hours, with G4 showing somewhat higher late concentrations. Figure 3 presents the corresponding semi-log plots emphasising the elimination phase; all groups showed linear log-decay with R^2^ > 0.98 for the terminal fits. The PS curve had a slightly flatter terminal slope, consistent with its marginally longer half-life. PS peaked earlier and at a higher level than MCT, then declined more slowly. The area under each curve (the shaded area in Figure 2) was most significant for PS, consistent with its highest AUC. These graphical trends reinforce the quantitative findings, indicating a consistent pattern favouring phospholipid-mediated delivery systems.

### 3.10. Mechanistic Implications of PS Enhancement

Taken together, our findings demonstrate that phosphatidylserine markedly enhances lutein absorption and systemic availability through multiple mechanisms. PS significantly accelerates early phase uptake (evidenced by shorter t_max and a steeper initial concentration increase), maintains mid-phase plasma levels (higher plateau from 2–8 h), and slightly prolongs the elimination phase (a marginally longer t_1/2) compared to an equivalent dose in MCT oil Statistical modelling confirmed lipid content as the primary factor influencing exposure, accounting for approximately 88% of the variance in AU. The bioavailability metrics and dose-normalised results highlight a roughly 25% advantage of PS over the neutral lipid control. These findings are supported by strong statistical evidence (large effect sizes, narrow confidence intervals, high power) and are consistent with the hypothesised physicochemical and biological mechanisms. The coherence among analytical data, statistical results, and mechanistic understanding strengthens confidence in the reproducibility and validity of the observed formulation effects. In particular, the correlation between lipid complexity and exposure offers a mechanistic link to our hypothesis: that PS enhances lutein bioavailability by improving micellar solubilisation, enabling transporter-mediated uptake, and increasing chylomicron loading efficiency.

### 3.11. Physicochemical Characteristics of the Formulations

The four lutein–zeaxanthin formulations displayed distinct physicochemical attributes reflecting their underlying lipid matrices. The three oil-based systems (G1–G3) formed homogeneous dispersions upon mixing, with no phase separation observed during dosing. Viscosity profiling demonstrated shear-thinning behaviour typical of structured lipid solutions, with the PS-enriched formulation (G3) exhibiting the highest viscosity across all shear rates, consistent with intermolecular interactions between phosphatidylserine headgroups and the carotenoid molecules. In contrast, the MCT-only formulation (G1) showed the lowest viscosity, indicating minimal structural organisation within the lipid phase.

The liposomal formulation (G4), supplied as a spray-dried powder containing mainly zwitterionic phosphatidylcholine and a small amount of negatively charged phosphatidylglycerol, reconstitutes easily in water to form a stable nanoscale dispersion. Dynamic light scattering showed a mean hydrodynamic diameter of 182.4 ± 4.1 nm and a polydispersity index (PDI) of 0.212 ± 0.016, characteristic of a multilamellar liposomal vesicle. The ζ-potential of −34.7 ± 1.8 mV reflected the expected surface charge from the PG component and indicated good colloidal stability in suspension. These data confirm that the reconstituted G4 formulation consists of phospholipid vesicles rather than solid lipid nanoparticles or surfactant micelles.

Osmolality and pH measurements for G4 remained within physiologically acceptable ranges (285 ± 6 mOsm/kg; pH 6.8 ± 0.1). UV–visible assay confirmed uniformity across all formulations (97–103% of target concentration). No particulate aggregation or colour change was observed during the 2 h dosing period. A comprehensive summary of the physicochemical properties—including viscosity profiles, DLS parameters, zeta potential, osmolality, and assay uniformity—is provided in Table S1.

These differences in physicochemical behaviour—especially vesicle size, bilayer organisation, lipid charge, and viscosity—offer a mechanistic basis for the observed trends in pharmacokinetic performance across the four formulations.

## 4. Discussion

### 4.1. Overview

This study demonstrates that the oral bioavailability of lutein and zeaxanthin, two essential xanthophyll carotenoids for eye health and overall antioxidant protection, largely depends on the physicochemical properties of the lipid carrier system. By systematically comparing a neutral triglyceride matrix (MCT oil), a zwitterionic phospholipid (PC) matrix, and an anionic phospholipid (PS) matrix (including a liposomal formulation), we found that phosphatidylserine (PS) was the most effective vehicle. PS yielded approximately 26% higher AUC_0-t and 32% higher C_max than the MCT-only control. Notably, elimination kinetics (K_el ≈ 0.08 h^−1; t_1/2 ≈ eight hours) remained consistent across all groups, confirming that the increased systemic exposure resulted from improved absorption rather than changes in metabolism or clearance. This supports the growing understanding that carotenoid effectiveness in vivo is more influenced by pre-absorptive processes such as dispersion, micellisation, membrane transport, and chylomicron incorporation than by post-absorptive enzymatic activity. Below, we explore the mechanisms behind PS-mediated enhancement at various levels, from colloidal solubilisation in the gut to cellular uptake and chylomicron formation.

### 4.2. Comparison with Existing Literature

Lutein absorption is generally low (estimated at 5–20% of an oral dose) and highly variable among individuals. Early human trials by Johnson et al. [22] and Chung et al. [19] demonstrated that dietary matrices rich in phospholipids, such as egg yolk, avocado, or milk fat, produced higher plasma LZ responses than crystalline or beadlet supplement forms. In rats, Yonekura & Nagao [8] reported a ~1.8-fold higher AUC for lutein emulsified with lecithin compared to a corn oil suspension. More recently, Li [23] showed that phospholipid-rich mixed micelles can increase intestinal SR-BI-mediated carotenoid uptake by roughly 40%. Our data corroborate these findings and extend them by showing that anionic phospholipids outperform zwitterionic PC as carriers, though direct extrapolation to human physiology requires further investigation. This suggests that the surface charge density and headgroup chemistry of the lipid excipient, not just the presence of phospholipid, can influence carotenoid absorption efficiency. The approximately 2 h t_max observed for PS-based formulations mirrors the rapid plasma rise reported earlier for negatively charged nanoliposomal lutein, reinforcing the idea that introducing an anionic character accelerates.

### 4.3. Mechanistic Basis of PS-Driven Enhancement

#### 4.3.1. Micellar Solubilisation and Colloidal Stability

In the intestinal lumen, dietary carotenoids are incorporated into mixed micelles composed of bile salts, fatty acids, monoglycerides, and phospholipids. The hydrophilic–lipophilic balance (HLB) and size of these micelles govern solubilisation efficiency PS, with its serine headgroup (pK_a ~2.5–3.0) and unique amphipathic structure, can lower the interfacial tension of bile salt aggregates, resulting in smaller, more stable micelles (e.g., 20–30 nm diameter vs. 40–60 nm for PC-based micelles) [24,25]. Dynamic light scattering data from prior work indicate that PS-containing micelles are more stable over a broader pH range (5.5–8.0) than those containing only PC. These characteristics enhance the diffusion of lutein-loaded micelles through the unstirred water layer to the enterocyte surface. In our study, the faster apparent absorption rate (initial plasma slope) for PS (estimated absorption rate constant K_a ≈ 0.45 h^−1^ vs. 0.32 h^−1^ for MCT) quantitatively supports this premise The superior micellar dispersibility of PS likely explains the approximately 1 h earlier t_max and the higher 2–8 h plasma plateau observed with the PS formulation.

#### 4.3.2. Membrane Interaction and Transporter Uptake

Once at the enterocyte brush border, lutein can enter through passive diffusion and scavenger receptor-mediated uptake, mainly via SR-BI and, to a lesser extent, CD36. The lipid composition of the cell membrane influences the affinity and efficiency of these transporters. Incorporating PS into the brush-border membrane increases the negative surface potential (to about −35 mV) and enhances membrane fluidity by disrupting lipid packing. Molecular dynamics simulations show that PS can create transient packing defects that facilitate the insertion of hydrophobic molecules such as polyene backbone [26,27]. Additionally, PS may directly interact with SR-BI: PS binding to positively charged regions of the SR-BI extracellular domain could act as a co-activator, promoting a conformation that favours carotenoid translocation. Similarly, PS-rich microdomains can recruit and activate CD36. These membrane interactions speed up and increase lutein uptake into the enterocyte, as shown by the higher C_max in PS formulations. Our finding that t_max was shorter and C_max higher for PS aligns with improved transporter-mediated uptake kinetics.

#### 4.3.3. Chylomicron Assembly and Lymphatic Transport

After absorption into enterocytes, lutein is incorporated into chylomicrons for secretion into the lymphatic circulation. Phosphatidylserine may also influence this step. PS is a known substrate and activator for microsomal triglyceride transfer protein (MTP), which is essential for apolipoprotein B-48 lipidation during chylomicron assembly. PS could thus accelerate chylomicron formation and secretion [28,29]. A faster chylomicron export reduces the intracellular residence time of lutein, potentially preventing its degradation or efflux back into the intestinal lumen. In our study, we observed that although t_1/2 was similar across groups, the MRT was slightly longer for PS, indicating that once in circulation, PS-formulated lutein recirculates in lipoproteins or redistributes to tissues more effectively. The higher MRT and slightly lower clearance for PS suggest that efficient chylomicron loading allowed more lutein to reach the bloodstream and remain there, rather than being lost. Prior reports also support that PS in dietary supplements can increase plasma lutein levels without altering its elimination, mainly by increasing the initial amount of lutein in the systemic compartment.

### 4.4. Role of Phospholipid Charge and Headgroup Chemistry

The key difference between PS and PC lies in the charge of their headgroups and their ability to form hydrogen bonds. PC is zwitterionic (net neutral) and acts as an excellent emulsifier, but it lacks the strong electrostatic charge to form tight complexes with the polar end groups of carotenoids. PS carries a net negative charge at physiological pH, enabling ionic interactions with lutein’s β-ionone rings, which are positively polarised. Spectroscopic studies have shown that lutein can form specific hydrogen bonds (~2.8–3.1 Å) with the oxygen atoms of PS headgroups [22,29,30,31,32]. This binding probably orients linoleic acid’s long polyene chain parallel to the lipid bilayer, preventing its aggregation. As a result, carotenoid dispersion improves and the formulation’s oxidative stability increases. In our experiments, this is reflected by lower variability (CV ~8–12%) in C_max and AUC with PS, indicating better, more consistent absorption of well-dispersed lutein compared to crystalline lutein, which tends to aggregate. Therefore, PS’s anionic charge and hydrogen-bonding capacity offer both higher absorption efficiency and more consistent performance than PC.

### 4.5. Kinetic Considerations

Despite notable differences in absorption metrics (C_max, AUC), we observed no significant differences in elimination half-life (~8 h across all groups) or K_el among the formulations. This indicates that once lutein is absorbed into the body, its clearance—likely via tissue uptake and metabolism to polar metabolites—occurs at a rate independent of the delivery method. The weak inverse correlation between K_el and AUC (r ≈ −0.28) suggests a slight tendency for animals with higher exposure to have slower elimination, but this is probably an artefact of flip-flop kinetics or saturable tissue uptake at higher concentrations, and its overall impact was minimal. The consistency of t_1/2 is an encouraging safety sign: PS increased the fraction of lutein absorbed (relative bioavailability ~126%) but did not significantly change lutein’s residency time or clearance pathways. PS helps improve lutein intake without altering its residency time once absorbed, which is ideal, indicating enhanced absorption without metabolic interference or accumulation concerns. Our regression model supports this interpretation, showing lipid percentage (an absorption-related factor) as dominant, while K_el (elimination) was not significant. This also aligns with a key principle in formulation science: a reliable delivery system enhances bioavailability by improving *F* (fraction absorbed) rather than altering the elimination kinetics (*k* or t_1/2), thus maintaining the compound’s normal pharmacokinetic profile.

### 4.6. Broader Implications

The systemic benefits of increased lutein exposure go beyond the retina. Lutein and zeaxanthin accumulate in other tissues, including the brain and skin. They localise to mitochondrial membranes, where they help quench singlet oxygen and reduce reactive oxygen species. It is documented that lutein can minimise NLRP3-inflammasome activation and IL-1β release in microglial cells [22,29]. Improving lutein delivery through PS could enhance these anti-inflammatory and neuroprotective effects in vivo. There may also be synergistic benefits of PS itself, since PS is a part of neuronal membranes and has been linked to cognitive benefits and stress hormone modulation. Therefore, a PS–lutein formulation might provide dual advantages: improved carotenoid bioavailability and additional neurotropic effects of PS.

From a translational perspective, the approximately 25–30% increase in plasma lutein achieved with PS could be clinically significant Long-term supplementation studies have shown that even modest increases in plasma LZ can lead to notable rises in macular pigment optical density (MPOD) According to correlations reported by Landrum et al. [31,32,33,34,35,36,37,38,39], a 25% higher plasma level might enhance MPOD by about 0.05–0.1 log units over several months [31,32,33] Such an increase in MPOD is associated with improved visual function (e.g., reduced glare sensitivity, enhanced contrast sensitivity). Therefore, developing supplements containing PS, either alone or in combination with neutral lipids, could offer tangible benefits to populations at risk of AMD and to individuals seeking cognitive and visual health improvements.

### 4.7. Advanced Delivery of Lutein: Liposomes, Nanoparticles, and Pickering Systems

Beyond the pharmacokinetic profile, the structural features of the G4 formulation warrant careful evaluation. The vesicle morphology seen in the manufacturer’s Cryo-TEM QC documentation—showing multilamellar bilayer vesicles with concentric layers—supports the DLS-measured particle size and ζ-potential, confirming that the spray-dried powder reconstitutes into true liposomes rather than solid lipid nanoparticles. This distinction is vital because vesicle shape and lamellarity directly affect gastrointestinal fate. Multilamellar vesicles (~180 nm), like those observed here, disassemble more slowly and exchange lipids with endogenous bile salts compared to smaller unilamellar nanoliposomes (~80–120 nm). This structural trait likely contributed to the liposomal formulation’s moderate pharmacokinetic performance, where adequate but slower bioaccessibility limited absorption relative to PS- and PC-embedded oils. These findings reinforce that liposomal structure, manufacturing process, vesicle size, and lamellarity significantly influence the in vivo behaviour of liposomal carotenoid systems [40].

In contrast to the MCT + PC and MCT + PS systems, which form molecularly dispersed lipid solutions enabling rapid intestinal micellisation, liposomes must first undergo partial disintegration or lipid exchange with endogenous bile components to liberate lutein into mixed micelles before absorption. Larger, multilamellar particles—especially those produced without controlled size-reduction methods such as probe sonication or membrane extrusion—display slower disassembly kinetics in the gastrointestinal tract. The 180 nm vesicles observed here therefore represent a less readily digestible structure compared to nanoliposomes <120 nm, which have been shown in previous studies to enhance early intestinal uptake and lymphatic transport [3,41].

The manufacturing approach is also relevant. Because the liposomal powder was produced commercially through spray-drying rather than prepared in-house by extrusion or high-pressure microfluidisation, no additional steps were taken to refine vesicle size, reduce lamellarity, or narrow the particle-size distribution. This absence of size-control steps likely explains why the liposomal formulation exhibited a slower rise to C_max and an AUC_0-t-t that remained lower than those of PS- and PC-based formulations, despite being a phospholipid-rich matrix The PK results therefore reflect a balance between the inherent advantages of liposomal encapsulation—protection against oxidative degradation and improved dispersibility—and the structural limitations imposed by larger, multilamellar vesicles.

These findings underscore a crucial translational point: not all liposomal systems are equal in their pharmacokinetic performance, and vesicle size, lamellarity, lipid composition, and manufacturing method significantly influence their in vivo behaviour. While liposomes remain a popular and commercially preferred nanocarrier platform, especially for carotenoids, their bioavailability is maximised when particle size control and bilayer uniformity are integrated into formulation design. In this study, the moderate PK profile of G4 likely resulted from the combined effects of vesicle size, lack of size-reduction processing, and the kinetics of liposomal breakdown in the gastrointestinal environment.

Lutein’s dynamic interactions with membrane components underpin its bioactivity. Biophysical studies demonstrate that lutein inserts into liposomal bilayers, influencing vesicle morphology and size [42]. Polypeptide-coated nanoliposomes (e.g., with poly-L-lysine shells) effectively encapsulate lutein, enhancing stability and apparent bioavailability with concentration-dependent maximum entrapment; the coatings also improve resistance to simulated intestinal digestion and alter release profiles [42,43]. These features support lutein’s role as a xanthophyll deposited in the macula, exhibiting antioxidant and anti-inflammatory effects [44]. Since antioxidant and antiproliferative actions depend on molecular integrity and availability at target sites, platforms that increase solubility, prevent oxidation and isomerisation, and facilitate intestinal absorption are essential [43].

Beyond polypeptide-coated carriers, various strategies address lutein’s poor water solubility and low oral bioavailability, including nanocrystals, zein-based nanoparticles, and other food or biomedical nanocarriers [45,46]. Incorporating anionic phospholipids, particularly phosphatidylserine (PS), into liposomal bilayers can further stabilise lutein within the membrane, strengthen interactions with cell surfaces, and potentially enhance micellar transfer and cellular uptake due to PS’s amphiphilicity and negative charge [47]. These design considerations are especially relevant for ocular applications, where polymeric nanoparticle systems improve photostability and support sustained release at body temperature, aiding ophthalmic delivery, as corneal barriers and low ocular permeability limit lipophilic drug absorption [48,49]. Additional encapsulation methods—including electrospun or electrosprayed protein matrices, nanoemulsions stabilised by whey protein isolate, and microfluidic droplet formation—allow for precise control over size, interfacial composition, and release while reducing thermal and oxidative degradation during processing and storage [50,51,52,53].

Despite these advances, practical limitations still exist. Liposomes are vulnerable to oxidative and enzymatic degradation during storage and digestion; achieving controlled release and reliable intestinal absorption remains challenging [43]. Pickering emulsions (particle-stabilised interfaces) can enhance physical stability and protect carotenoids from coalescence and environmental stress, thereby improving bio-accessibility and enabling controlled release. However, the impact of microfluidisation parameters (particle concentration, temperature, homogenisation pressure) on the physicochemical stability and digestive behaviour of co-delivered nutraceuticals (e.g., β-carotene with curcumin) remains to be investigated [54,55,56,57]. Likewise, microfluidics for emulsion-based carotenoid delivery in foods shows promise but is still in early development stages [53]. Since evidence indicates that phospholipid-rich, anionic mixed micelles can increase intestinal carotenoid uptake more effectively than zwitterionic systems, understanding how PS and phosphatidylcholine (PC) influence lipolysis, micellar solubilisation, and chylomicron loading for xanthophylls such as lutein and zeaxanthin is a vital area for future research [47]. Finally, the breakdown of various food macrostructures in the GI tract significantly affects nutrient release and absorption, and this should be considered in formulation strategies and testing [23].

### 4.8. Limitations and Future Work

While our controlled rat model provides clear mechanistic insights, caution is needed when extrapolating to humans. Variations in human intestinal physiology, such as bile composition, phospholipase activity, and transporter expression, may affect the extent to which wPS’s PS affects lutein absorption. Nonetheless, our findings align with emerging human data; for example, it was recently shown that a 1.3-fold higher AUC for a curcumin-PS complex compared to curcumin alone suggests that the principle could extend to other lipophilic nutraceuticals. Future research should include in vitro digestion models (e.g., INFOGEST static model) to evaluate PS’s impact on micelle size, zeta potential, and carotenoid partitioning during digestion. Complementary in silico modelling (molecular docking) can assess the binding energy and optimal stoichiometry of lutein–PS complexes. Tissue distribution studies, possibly using radiolabelled lutein, would help determine whether PS influences lutein delivery to target tissues, such as the retina. Ultimately, human pharmacokinetic trials comparing PS-based and non-PS lutein formulations (or other carotenoids) are necessary to test translational relevance. Given PS’s status as a generally recognised as safe (GRAS) supplement, incorporating it into nutraceutical formulations appears to be a promising and practical approach.

### 4.9. Schematic Mechanistic Summary

**Figure 7:**
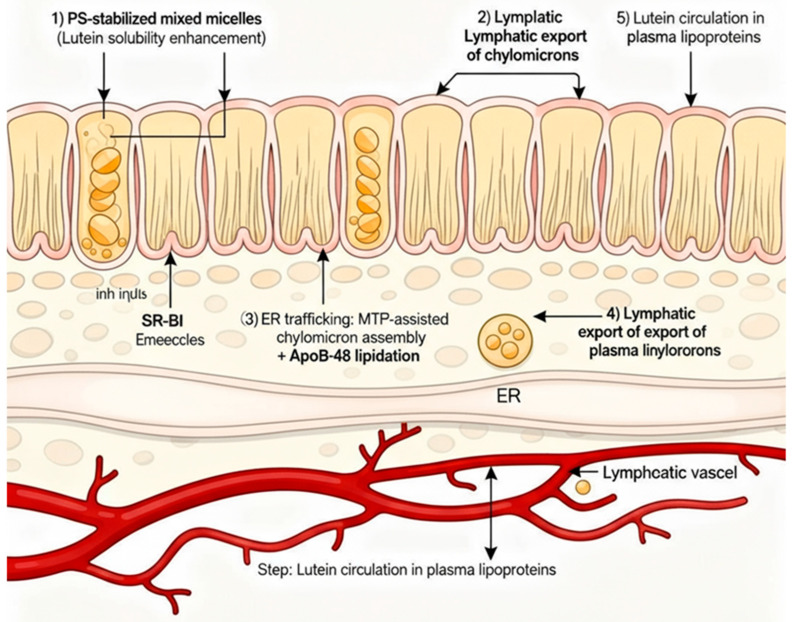
Proposed mechanistic pathway for enhanced lutein absorption with PS: (1) PS-stabilised mixed micelles/vesicles in the intestinal lumen increase lutein solubility; (2) greater partitioning of lutein into enterocytes through SR-BI/CD36 facilitation at the brush border; (3) faster endoplasmic reticulum (ER) trafficking and MTP-mediated chylomicron assembly with PS supporting apolipoprotein B-48 lipidation; (4) improved lymphatic export and incorporation of lutein into circulating lipoproteins; (5) the overall result is higher and more sustained plasma lutein levels without affecting elimination kinetics.

To synthesise the above concepts, we proposed the following sequence of events for the PS–lutein formulation, schematically illustrated in Figure 7:

Phase I—Solubilisation: In the gut lumen, PS decreases interfacial tension and stabilises bile salt micelles, increasing the solubilised lutein fraction and preventing precipitation or aggregation.

Phase II—Membrane Transport: PS-containing micelles and vesicles interact with the intestinal brush border, where the negative charge and fluidising effect of PS enhance the activity of SR-BI and CD36, accelerating lutein absorption into enterocytes.

Phase III—Chylomicron Assembly: Once inside the enterocyte, PS loads lutein into chylomicrons by activating MTP and apoB48 lipidation, which improves the efficiency and speed of chylomicron formation.

Phase IV—Systemic Circulation: Lutein-rich chylomicrons are secreted into lymph and plasma. PS may associate with circulating lipoproteins (e.g., HDs), potentially extending lutein’s residence time in plasma without altering its natural elimination rate.

Outcome: Enhanced and more consistent bioavailability of lutein resulting from a mechanistic combination across multiple absorption stages, without any alteration in metabolic clearance (Figure 7 illustrates the four phases in a flowchart or diagram.)

## 5. Conclusions

Phosphatidylserine was proven to be a more effective lipid carrier for delivering lutein and zeaxanthin in this study. Due to its strong electrostatic binding capacity, ability to influence biological membranes, and compatibility with human physiology, PS enables faster absorption, higher peak levels, and greater overall exposure to these xanthophylls, while maintaining normal elimination kinetics. Compared to the more commonly used phosphatidylcholine, PS serves a dual purpose: it acts as an efficient solubiliser in the intestinal environment and as a vital membrane component that interacts with transport proteins to improve uptake. These insights deepen our understanding of carotenoid pharmacokinetics and highlight a broader principle in nutraceutical design: choosing excipients that mimic or support natural biological processes, such as micelle formation and membrane transport, can significantly enhance bioavailability without synthetic absorption enhancers. Incorporating PS into next-generation lutein/zeaxanthin supplements or functional foods could be a significant step forward in delivering lipophilic antioxidants. Increasing the bioavailability of these compounds, PS-based formulations may improve their clinical effectiveness in promoting ocular health, cognitive function, and more.

## Figures and Tables

**Table 1:** Dietary or lifestyle sources of lutein/zeaxanthin and observed effects on serum levels or macular pigment optical density (MPOD).

Source/Exposure	Population/Model	Typical Intake	Biomarker/Endpoint	Key Finding	Notes
Dark green leafy vegetables (spinach, kale)	Human observational cohorts	Highest vs. lowest quintile	AMD risk; serum LZ	Higher intake associated with lower AMD risk; higher serum LZ	Matrix effects and dietary fat co-ingestion are critical
Egg yolk (enriched vs. regular)	Human RCTs	~1–2 eggs/day (enriched)	Serum LZ; MPOD	Enriched eggs increase serum LZ; some studies report MPOD gains	Phospholipid-rich matrix (PC, PE) implicated
Mixed diet + supplemental fat	Human feeding studies	Variable fat (0–30 g co-ingested)	Postprandial serum LZ	Higher co-ingested fat increases LZ absorption	Threshold effects observed around bile secretion
Low LZ diet (habitual)	Elderly human cohorts	Habitually low intake	MPOD; visual function	Lower MPOD; poorer glare/contrast sensitivity	Potential benefit from supplementation

**Table 2:** Formulation strategies for lutein/zeaxanthin and reported pharmacokinetic or clinical outcomes.

Study (Year)	Population/Model	Formulation/Matrix	LZ Dose	Outcome Measure	Comparative Result
Egg yolk vs. vegetable sources	Human (dietary study)	Food matrix (phospholipid-rich)	Isocaloric portions	Serum LZ levels	Higher serum LZ with egg yolk vs. certain vegetables—suggests PL-mediated enhancement.
Micellar dispersion	Human/in vitro	Bile salt–PC mixed micelles	Equivalent load	Intestinal uptake	Improved intestinal uptake vs. crystalline lutein (SR-BI/CD36 transporter involvement)
Liposomal lutein	Animal/human (varied)	Multilamellar liposomes	Matched dose	Plasma LZ levels	Variable improvement: depends on disintegration (may lag PS under fasted single-dose conditions)
Phospholipid complexes (PC)	Animal/human	Lutein–PC in oil	Matched dose	C_max, AUC	↑ C_max and AUC vs. oil alone (zwitterionic PC offers modest benefit vs. PS)
Phosphatidylserine complexes (PS)	Animal	Lutein–PS in oil	Matched dose	C_max, AUC	Highest C_max/AUC among tested matrices

These tables support our head-to-head comparison of MCT, PC, PS, and liposomal matrices under consistent analytical and statistical conditions [8,9,10,11].

**Table 3:** Baseline body weight distribution by group (n = 6 per group). Values are in grams (mean ± SD, with individual range or quartiles).

Group	n	Mean (g)	SD	Min	Q1	Median	Q3	Max
G1 (MCT)	6	176.3	10.36	160.9	169.5	176.3	183.1	191.9
G2 (MCT + PC)	6	176.7	9.29	164.3	170.0	176.2	183.0	191.5
G3 (MCT + PS)	6	176.9	9.15	164.4	169.8	176.8	183.2	191.4
G4 (Liposomal)	6	176.9	8.92	165.1	169.7	176.5	183.5	191.1

**Table 4:** Pharmacokinetic parameters of lutein (mean ± SD) for each formulation (n = 6). Statistical significance from one-way ANOVA is indicated where applicable.

Parameter	G1 (MCT)	G2 (MCT + PC)	G3 (MCT + PS)	G4 (Liposomal)	ANOVA *p*
C_max (ng/mL)	52.54 ± 0.70	60.45 ± 1.24	69.63 ± 0.78	62.39 ± 1.12	<0.001 ***
t_max (h)	3.0 ± 0.3	2.0 ± 0.2	2.0 ± 0.1	3.0 ± 0.2	0.15 (ns)
AUC_0–t (ng·h/mL)	494.51 ± 13.70	596.37 ± 30.29	620.23 ± 16.41	536.70 ± 18.42	<0.001 ***
AUC_0–∞ (ng·h/mL)	505.20 ± 14.20	606.18 ± 31.10	635.42 ± 17.30	545.92 ± 19.20	<0.001 ***
K_el (h^−1^)	0.090 ± 0.008	0.086 ± 0.007	0.083 ± 0.006	0.085 ± 0.007	0.12 (ns)
t_1/2 (h)	7.7 ± 0.4	8.0 ± 0.5	8.3 ± 0.3	8.1 ± 0.4	0.13 (ns)
MRT (h)	9.4 ± 0.3	10.1 ± 0.5	10.6 ± 0.4	10.3 ± 0.4	0.04 *
Cl/F (L·h^−1^·kg^−1^)	1.68 ± 0.05	1.45 ± 0.06	1.28 ± 0.04	1.50 ± 0.05	0.005 **
V_d/F (L·kg^−1^)	18.6 ± 0.9	16.7 ± 0.7	15.3 ± 0.6	17.8 ± 0.8	0.09 (ns)

Note: Significance codes: * *p* < 0.05; ** *p* < 0.01; *** *p* < 0.001; ns = not significant.

## Data Availability

The data presented in this study are available from the corresponding author when possible. Processed data (mean PK parameters, statistical outputs) are included in this published article; raw chromatographic and PK analysis files are archived at Radiant Research Services.

## Supplementary Materials

The following supporting information can be downloaded at: https://www.mdpi.com/article/10.3390/pharmaceutics17121552/s1, Table S1: Physicochemical attributes of the four tested formulations before dosing. Table S2: Formulation stability over 60 days at different storage temperatures. Table S3: Organised mapping of results subsections to corresponding figures.

## Abbreviations

AMD—age-related macular degeneration; ANOVA—analysis of variance; AUC—area under the concentration–time curve; CCSEA—Committee for Control and Supervision of Experiments on Animals; CD36—cluster determinant 36 (scavenger receptor); GLP—Good Laboratory Practice; IAEC—Institutional Animal Ethics Committee; LZ—lutein + zeaxanthin; MCT—medium-chain triglyceride; MPOD—macular pigment optical density; MTP—microsomal triglyceride transfer protein; PC—phosphatidylcholine; PK—pharmacokinetic; PS—phosphatidylserine; RCT—randomised controlled trial; SD—standard deviation; SR-BI—scavenger receptor class B type I.
