# Supplementary material for: Comparative Pharmacokinetics of Lutein and Zeaxanthin from Phospholipid, Liposomal, and MCT Formulations in SD Rats
Source: Pharmaceutics. 2025 Dec 2;17(12):1552. doi: 10.3390/pharmaceutics17121552 (PMC12736751; doi:10.3390/pharmaceutics17121552)
Supplement: Supplementary file 1 [file pharmaceutics-17-01552-s001.zip › pharmaceutics-3961890-supplementary.pdf]

Table S1. Physicochemical attributes of the four tested formulations before dosing.

Values expressed as mean ± SD (n = 3). G1–G3 are oil dispersions; G4 is an aqueous liposomal dispersion.

**Parameter**	**G1: MCT**	**G2: MCT + PC**	**G3: MCT + PS**	**G4: Liposomal (Reconstituted)**
**DLS Z-average (nm)**	— (non-colloidal)	— (non-colloidal)	— (non-colloidal)	**182.4 ± 4.1**
**PDI**	—	—	—	**0.212 ± 0.016**
**Zeta potential (mV)**	—	—	—	**–34.7 ± 1.8**
**Viscosity @10 s^{-1} (mPa·s)**	68.2 ± 3.1	72.5 ± 4.0	85.9 ± 3.8	—
**Viscosity @50 s^{-1} (mPa·s)**	52.1 ± 1.8	61.3 ± 2.9	70.5 ± 3.0	—
**Viscosity @100 s^{-1} (mPa·s)**	45.3 ± 2.2	55.7 ± 2.1	63.4 ± 2.6	—
**Osmolality (mOsm/kg)**	—	—	—	285 ± 6
**pH (aqueous phase)**	—	—	—	6.8 ± 0.1
**UV-Vis assay @ 445 nm**	98–102% of target	99–103%	98–101%	97–102%

Notes:

- Oils (G1–G3) do not produce DLS/PDI values; only the liposomal dispersion (G4) does.
- MCT + PS (G3) shows the highest viscosity, consistent with stronger polar lipid interactions.

Table S2. Formulation stability over 60 days at different storage temperatures.

Stability endpoints: retention of lutein (%), change in particle size/PDI (G4), visual appearance

**Condition**	**G1: MCT**	**G2: MCT + PC**	**G3: MCT + PS**	**G4: Liposomal (Reconstituted)**
**–70 °C (baseline in manuscript)**	>98% potency; no changes	>98% potency	>98% potency	Size: 182 → 184 nm; PDI unchanged
**4 °C (60 d)**	95% potency; no phase separation	96% potency; slight thickening	97% potency; unchanged	Size: 182 → 191 nm; PDI 0.21 → 0.25
**25 °C (60 °F)**	89% potency; mild colour deepening	91% potency	**94% potency; most stable**	Size: 182 → 229 nm; PDI rises to 0.31; mild aggregation
**Visual Appearance**	Clear yellow oil	Slightly hazy lipid layer	Clear, stable	Slight opalescence at 25 °C

Table S3. Organised mapping of results subsections to corresponding figures

Results Section	Description	Corresponding Figure(s)
**3.1 Physicochemical Characterisation**	DLS, PDI, ζ, viscosity, osmolality	**Table S1**
**3.2 Plasma Concentration Profiles**	Linear and semi-log PK curves	**Figure 2, Figure 6**
**3.3 PK Parameters**	Cmax, tmax, AUC, clearance	**Figure 3, Figure 4**
**3.4 Statistics**	ANOVA, effect sizes, variability	Within text (referenced)
**3.5 Correlation Analyses**	Cmax vs AUC, lipid% vs AUC	**Figure 5**
**3.6 Extended Bioavailability**	GMR calculations	Figure references retained
**3.9–3.10 Mechanistic Interpretation**	PS vs PC vs MCT	Figure 7 (schematic)
